# Heterogeneous Effects of Direct Hypoxia Pathway Activation in Kidney Cancer

**DOI:** 10.1371/journal.pone.0134645

**Published:** 2015-08-11

**Authors:** Rafik Salama, Norma Masson, Peter Simpson, Lina Katrin Sciesielski, Min Sun, Ya-Min Tian, Peter John Ratcliffe, David Robert Mole

**Affiliations:** The Henry Wellcome Building for Molecular Physiology, Nuffield Department of Medicine, University of Oxford, Oxford, United Kingdom; Duke University Medical Center, UNITED STATES

## Abstract

General activation of hypoxia-inducible factor (HIF) pathways is classically associated with adverse prognosis in cancer and has been proposed to contribute to oncogenic drive. In clear cell renal carcinoma (CCRC) HIF pathways are upregulated by inactivation of the von-Hippel-Lindau tumor suppressor. However HIF-1**α** and HIF-2**α** have contrasting effects on experimental tumor progression. To better understand this paradox we examined pan-genomic patterns of HIF DNA binding and associated gene expression in response to manipulation of HIF-1**α** and HIF-2**α** and related the findings to CCRC prognosis. Our findings reveal distinct pan-genomic organization of canonical and non-canonical HIF isoform-specific DNA binding at thousands of sites. Overall associations were observed between HIF-1**α**-specific binding, and genes associated with favorable prognosis and between HIF-2**α**-specific binding and adverse prognosis. However within each isoform-specific set, individual gene associations were heterogeneous in sign and magnitude, suggesting that activation of each HIF-**α** isoform contributes a highly complex mix of pro- and anti-tumorigenic effects.

## Introduction

Hypoxia is strongly associated with adverse prognosis in cancer and hypoxia signaling pathways are commonly activated during cancer development[1–4]. This is exemplified by clear cell renal cancer (CCRC) in which inactivation of the von Hippel-Lindau tumor suppressor (pVHL) is a common and early event[5–7]. pVHL is the recognition component of an E3 ubiquitin ligase complex that targets hypoxia inducible factor (HIF) **α**-subunits for degradation by the ubiquitin-proteasome pathway, and inactivation of pVHL results in constitutive activation of the HIF transcriptional pathway[8]. HIF transcriptional targets include many with functions that associate with common phenotypic characteristics of cancer (e.g. enhanced angiogenesis, dysregulated energy metabolism, increased cell motility), and it is commonly assumed that oncogenesis is driven largely by the aggregation of the positive effects of HIF activation on such processes.

Transcriptional activation by HIF is principally mediated through binding of HIF **α**/**β** heterodimers to a core consensus sequence (RCGTG) in hypoxia response elements (HREs)[9]. Although the two best-characterized HIF-**α** subunits, HIF-1**α** and HIF-2**α**, manifest similar domain architecture, and indistinguishable DNA-binding sequences[10], they transactivate distinct targets and show contrasting tumorigenic roles[11,12]. Surprisingly, strong up-regulation of HIF following inactivation of VHL in CCRC is associated with an unusual bias in HIF isoform expression, towards HIF-2**α**[13]. Several lines of investigation indicate that this is functionally important in the development of CCRC. Genome-wide association studies identified polymorphic variants that affect susceptibility to CCRC at the HIF-2**α**, but not HIF-1**α**, locus and at loci within the HIF-2**α** transcriptional pathway[14]. Conversely, HIF-1**α** gene dosage is commonly reduced in CCRC by loss of chromosome 14q [15], and the HIF-1**α** gene, but not the HIF-2**α** gene, is subject to a small but significant excess of inactivating mutations[16]. Furthermore, HIF-2**α** but not HIF-1**α**, can over-ride the tumor suppressor activity of pVHL in experimental tumor systems[17,18]. Specifically, re-expression of HIF-1**α** in CCRC lines that lack wild-type HIF-1**α** slows growth, whilst overexpression of HIF-2**α** accelerates growth in tumor xenografts[11,15].

These observations challenge the paradigm that general activation of HIF signaling drives oncogenesis through activation of a small number of discrete transcriptional targets, and suggest a more complex interface. For instance, pan-genomic analyses revealed hundreds to thousands of direct HIF transcriptional targets[10,19–22]. Since HIF-1**α** and HIF-2**α** may potentially compete for binding to HIF-1β or for occupancy of HREs, or may bind distinct sets of transcriptional targets, it is unclear how the isoform specific manipulation of HIF-**α** impacts on the transcription patterns associated with CCRC.

To study this we performed detailed ChIP-seq and RNA-seq analysis of HIF-**α** isoform binding site occupancy and gene expression in the pVHL-defective CCRC 786–0 cell line, following re-expression of HIF-1**α** or overexpression of HIF-2**α**, and related these findings to prognostically associated patterns of gene expression in human CCRC tumors[6]. Our findings reveal large numbers of discrete isoform-specific HIF-**α** binding sites that manifest an isoform-specific genomic architecture, activate distinct patterns of gene expression and associate with contrasting prognostic gene expression patterns in clinical CCRC. However, although clear overall associations were observed between HIF-1**α**-associated genes and good clinical prognosis and between HIF-2**α**-associated genes and poor clinical prognosis, this dichotomy was incomplete. At the level of individual genes, the effects were heterogeneous in both sign and size of effect, suggesting that even within this defined context, each HIF-**α** isoform has potentially both pro- and anti-tumorigenic effects.

## Materials and Methods

### Laboratory methods

#### Cell Culture

786-O and HEK293T cells were purchased from ATCC (http://www.lgcstandards-atcc.org) and grown in Dulbecco modified Eagle medium supplemented with 10% fetal calf serum, 2 mM L-glutamine, 100U penicillin and streptomycin 50 U/ml (v/v) (Sigma-Aldrich).

#### Production of 786–0 HIF-1α/HIF-2α cells

HIF-1**α** and HIF-2**α** cDNA sequences[11] were first cloned into pRRL.IRES.EGFP (kind gift from Kamil Kranc, Glasgow) to generate bicistronic vectors. These were then co-transfected with pCMV-dR8.2 and pCMC-VSVG into HEK293T cells and the resultant viral particles were isolated by centrifugation and ultrafiltration. 786–0 cells (ATCC) were then transduced with either control (pRRL), HIF-1**α** (pRRL-HIF-1**α**) or HIF-2**α** (pRRL-HIF-2**α**) expressing virus.

#### ChIP-seq

Single ChIP-seq analyses were performed as previously described[19]. Chromatin was immunoprecipitated using rabbit polyclonal antisera to HIF-1**α** (PM14), HIF-2**α** (PM9)[23], HIF-1β (NB-100-110, Novus Biologicals, UK) or pre-immune serum as control.

#### PolyA+ selected RNA-seq

Total RNA was prepared in triplicate using the mirVana miRNA Isolation Kit (Ambion; Life Technologies Ltd, Paisley, UK) and treated with DNaseI (TURBO DNA‐free, Ambion). PolyA+ RNA libraries were then prepared using the ScriptSeq v2 RNA‐Seq kit (Epicentre, Madison, WI, USA).

#### High-throughput sequencing

All libraries were prepared according to Illumina protocols and sequenced on the HiSeq 2000 platform (Illumina, San Diego, CA, USA).

#### Accession codes

ChIP-seq and RNA-seq data are available from Gene Expression Omnibus (GSE67237).

### Bioinformatic analysis of ChIP-seq data

#### Initial Analysis

Illumina adaptor sequences were trimmed using Trimgalore (0.3.3) and reads were aligned to Genome Reference Consortium GRCh37 (hg19) using BWA (0.7.5a-r405). Low quality mapping was removed (MapQ < 15) using SAMtools (0.1.19)[24] and reads mapping to Duke Encode black list regions (http://hgwdev.cse.ucsc.edu/cgi-bin/hgFileUi?db=hg19&g=wgEncodeMapability) were excluded using BEDTools (2.17.0)[25]. Duplicate reads were marked for exclusion using Picard tools (1.106) (http://picard.sourceforge.net/). Read densities were normalized and expressed as reads per kilobase per million reads (RPKM)[26]. One million random non-overlapping regions selected from ENCODE DNase Cluster II peaks (http://hgdownload.cse.ucsc.edu/goldenPath/hg19/encodeDCC/wgEncodeRegDnaseClustered/) were used as a control.

#### Peak Calling

ChIP-seq peaks were identified using T-PIC (Tree shape Peak Identification for ChIP-Seq)[27] and MACS (Model-based analysis of ChIP-Seq)[28] in control mode. Peaks detected by both peak callers were filtered quantitatively using the total count under the peak to include only peaks that were above the 99.99th percentile of random background regions selected from the ENCODE DNASE II cluster (p-value < 0.0001).

#### De-novo Motif Analysis

Sequences flanking each peak summit (±150bp) were repeat-masked using RepeatMasker 4.0.3 (http://www.repeatmasker.org). De-novo motifs were identified using Meme-chip (4.9.1)[29] and matched, using the TomTom module, to known transcription factor motifs in the 2009 JASPAR core database[30].

#### Principal Component Analysis (PCA)

Binding sites for all subunits included in the PCA were merged into one binding set. PCA was performed using Singular Value Decomposition (Prcomp, R 3.1.1 –stats library, http://cran.r-project.org) for both individual binding sites and for the entirety of the ChIP-seq signal for each subunit. Biplots were generated in R.

#### Heat maps

Binding site heat maps were generated using Ngsplot (2.08)[31] with the parameters: FL = 50, MW = 5, RZ = 1, SC = 0–1, MQ = 15.

#### Motif Likelihood

Each HIF-**α** binding region (summit ±150bp) was scanned for the HRE (Jaspar/MA0259.1) and the AP-1 (Jaspar/MA0491.1) binding motifs using position weight matrices (PWMs) retrieved from the 2009 JASPAR Core database[30]. The normalized likelihood ratio (NLR) for each motif was calculated according to Eq 1. The maximum normalized likelihood ratio for each binding region was reported for both the HRE and AP-1 motif as given by Eq 2.

$${NLR}_{j}=\frac{1}{L}\sum_{i=1}^{L}\frac{PWM\left(b,i\right)}{B\left(b\right)},$$ Equation (1)

$$P_{NLR}=\text{max}\left({⋃}_{j=1}^{W-L}\;{NLR}_{j}\right),$$ Equation (2)

Where ***j*** is the position in the peak, ***i*** is the position in the motif, ***L*** is the length of the motif, ***PWM(b*,*i)*** is the PWM value at *i* for the corresponding base *b*, ***B(b)*** is the background weight for the corresponding base calculated over a million random DNAse sites and ***W*** is the peak width.

### Bioinformatic analysis of RNA-seq data

#### Initial Analysis

Adapter sequences were trimmed as above. Reads were then aligned to GRCh37 using Tophat 2.0.8b (http://ccb.jhu.edu/software/tophat/index.shtml) and bowtie 1.0.0 (http://bowtie-bio.sourceforge.net/index.shtml) and non-uniquely mapping fragments excluded using SAMtools (0.1.19)[24]. Total read counts for each UCSC defined gene were extracted using HTSeq (0.5.4p3)[32] with ‘intersection-strict’ mode and significantly regulated genes were identified using DESeq2 (ref. [33]).

#### Gene Set Enrichment Analysis (GSEA)

GSEA enrichment analysis used 10000 permutations, weighted enrichment score and pre-ranking of genes[34]. Both differential expression significance according to DESeq2 and fold-difference between the two conditions were used to rank genes[35] (Eq 3).

$$\pi_{i}=\;\varphi_{i}\left(-{log}_{10}{pv}_{i}\right),$$ Equation 3

Where **φ**_**i**_ is the log2 fold-change and ***pv***_***i***_ is the p-value for gene *i*.

#### The Cancer Genome Atlas (TCGA) GSEA

Clinical and RNA-seq V2 data for Kidney Renal Clear Cell Carcinoma (KIRC) patients (https://tcga-data.nci.nih.gov/tcga/) [6] was collated. Patients with missing clinical data or multiple RNA-seq datasets were excluded. Normalized mRNA counts were used as provided. Patients were divided into two groups using a prognostic score, adding 1 for each of; age over 60, pathologic stage 3 or 4, presence of metastasis, or patient deceased. Good prognosis patients scored 0 or 1 and bad prognosis patients scored 2, 3 or 4. The differential expression of each gene between the patient groups was assessed by the Likelihood Ratio Test for negative binomial fitted models using glm.nb in R (3.1.1). Genes were ranked for GSEA based on combined significance and fold-change as above.

#### Disease Annotation

Binding site enrichment was calculated using -log10 of the binomial test FDR (q-value) by GREAT (Genomic Regions Enrichment of Annotations Tool[36]) 2.0.2. Cancer subtypes with no enrichment in either ChIP-seq dataset were removed.

#### Binding Gene Predictor

Genes within 10-kb of sites with at least 3-fold difference between HIF-1**α** and HIF-2**α** binding were selected to design a gene predictor using Supervised Principle Component Analysis (SPCA)[37,38]. First, HIF-binding genes demonstrating univariate significance (p<0.05) in predicting patient prognosis (Cox proportional hazard model) were selected. For each set of genes (those binding re-introduced HIF-1**α** or overexpressed HIF-2**α**) Singular Value Decomposition (SVD) across all the TCGA patients was used to assign gene weights[37,38]. Each gene predictor was validated using ‘leave-one-out’ cross-validation, employing 414 patients at a time to generate the gene predictor and then predict the survival of each left-out patient. The final significance and hazard ratio of the patient’s risk predictor was assessed similar to univariate analysis.

## Results

Firstly, to define the HIF isoform specific activities that are associated with contrasting effects on growth in CCRC-derived VHL-defective 786–0 cells, pools of cells were infected with viruses expressing empty vector (VA), wild-type HIF-1**α**, or wild-type HIF-2**α**, and pan-genomic patterns of HIF-binding and gene expression were analyzed by ChIP-seq and RNA-seq. HIF-1**α** and HIF-2**α** infections achieved roughly equal mRNA levels that were approximately 10-fold higher than the endogenous HIF-2**α** mRNA level (S1A Fig). Similarly, HIF-2**α** protein levels were approximately 8-fold higher in the HIF-2**α** infected cells than in the control cells, whilst HIF-1**α** protein levels were 15-20-times higher in HIF-1**α** infected cells than in another CCRC cell line (RCC4), which expresses full-length HIF-1**α** (S1B Fig). In control cells, a total of 1719 peaks binding endogenous HIF-2**α** were identified. As anticipated, these sites show a high level of concordance with HIF-1**β** signal, consistent with binding of a HIF-2**α**/1**β** heterodimer (S2 Fig).

Our RNA-seq analysis confirmed previous findings[15] that, whilst 786–0 cells do not express wild-type HIF-1**α**, they do express several truncated and/or fusion transcripts encompassing HIF-1**α** exons 1–9, but not more distal sequences (S3 Fig), and are predicted to be incapable of encoding a transcriptionally active HIF-1**α**. Re-expression of full-length wild-type HIF-1**α** in 786–0 cells has a negative effect on growth as tumor xenografts in mice. We therefore began by examining pan-genomic patterns of DNA binding for HIF-1**α**, HIF-1**β** and HIF-2**α** in such cells, and compared these with control 786–0 cells.

### Re-expression of HIF-1α does not antagonize HIF-2α binding

**Fig 1:**
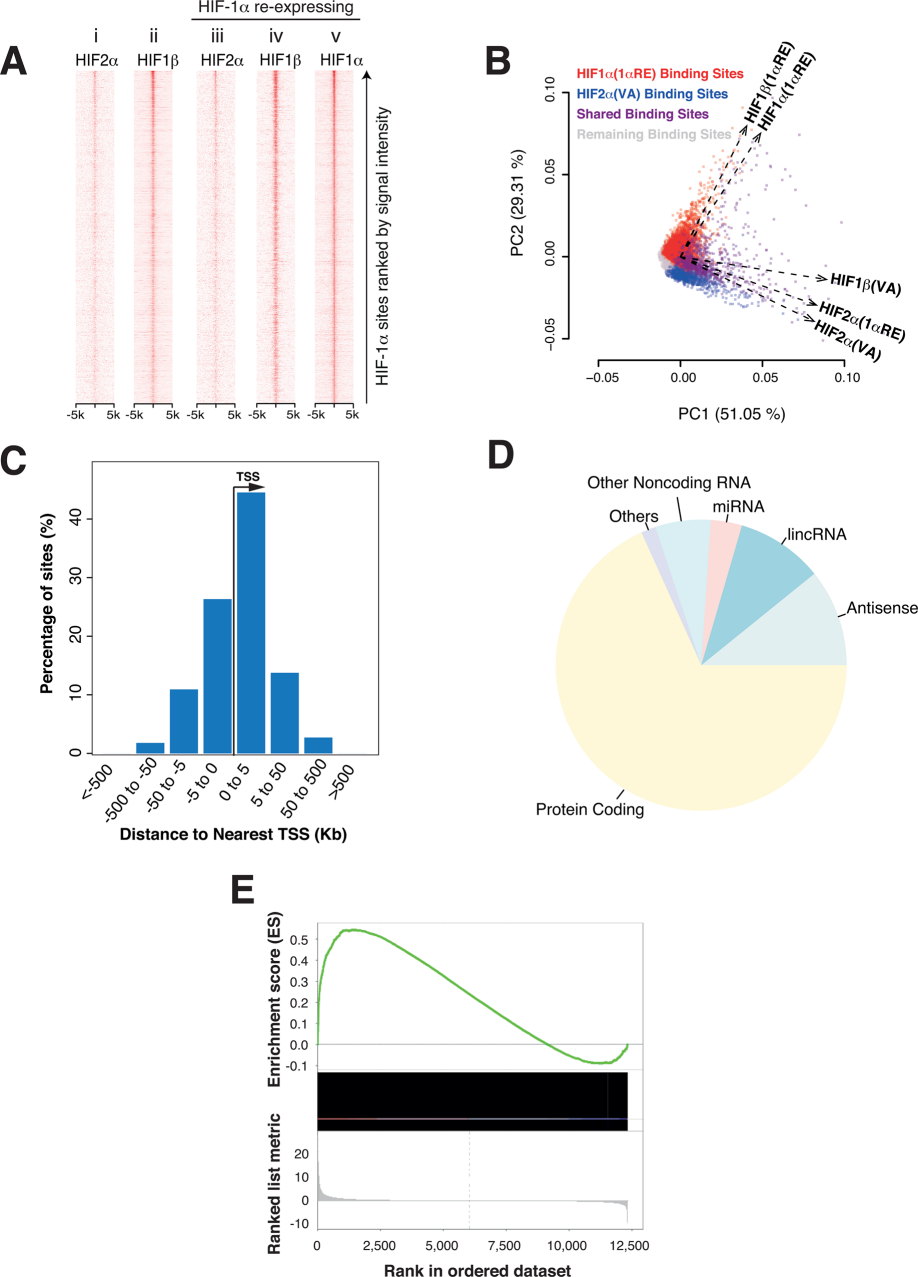
HIF-1α re-expression in 786-O cells. (A) HIF-1**α** binding sites in the HIF-1**α** re-expressing cells were identified by peak calling and ranked on the vertical axis according to signal intensity. Heat maps of these sites (±5kb on horizontal axis) showing ChIP-seq read density for the indicated HIF subunits were generated for both the control cells (i, ii) and the HIF-1**α** re-expressing cells with HIF-1**α** re-introduced (iii-v). The pattern of HIF-2**α** binding is minimally affected by the re-expression of full-length HIF-1**α** (compare i and iii). Sites binding re-expressed HIF-1**α** are largely co-occupied by HIF-1**β** (compare iv and v). (B) Biplot showing Principal Component Analysis (PCA) of ChIP-seq signal intensity (RPKM values) for both individual binding sites (dots) and HIF-subunits (vectors) across all HIF-binding sites identified in control cells and in HIF-1**α** re-expressing cells. Sites binding endogenous HIF-2**α** in control cells are shown in blue while sites binding re-expressed HIF-1**α** are shown in red, sites binding both are colored purple and the remaining sites are shown in grey. PCA for each subunit shows high co-variance between HIF-2**α** binding in the control cells and in the HIF-1**α** re-expressing cells (compare HIF2**α**(VA) and (HIF2**α**(1**α**RE)). This indicates only minimal change in the HIF-2**α** binding as a consequence of the HIF-1**α** re-expression. Conversely, the HIF-1**β** vector changes dramatically with HIF-1**α** re-expression (compare HIF1**β**(VA) with HIF1**β**(1**α**RE)) and aligns closely with the vector for re-expressed HIF-1**α** (HIF1**α**(1**α**RE)). The individual binding sites in the control and HIF-1**α** re-expressing cells (blue and red dots) aligned closely with their respective PCA vectors. (C) Histogram of the distance to nearest transcription start site (TSS) for HIF-1**α** binding sites in cells re-expressing HIF-1**α**. (D) HIF-1**α** binding sites in the re-expressing cells were categorized according to the class (Ensemble) of the nearest gene. The relative frequency of each class is show by pie chart. (E) Gene set enrichment analysis (GSEA) for the set of genes nearest to HIF-1**α** binding sites when genes are ranked according to fold-change and significance in mRNA expression following re-expression of HIF-1**α** (horizontal axis).

Since HIF-1**α** and HIF-2**α** both dimerize with HIF-1**β**, and recognize a similar consensus DNA sequence, but have opposing effects in tumor xenograft growth in 786–0, we first considered the possibility that re-expressed HIF-1**α** might antagonize HIF-2**α** DNA-binding, either through direct competition for binding sites or through competition for HIF-1**β**. Somewhat surprisingly, genome-wide analysis revealed that HIF-2**α** binding was little affected by re-expression of HIF-1**α** (Fig 1A, compare i and iii). Consistent with this, principal component analysis (PCA) of HIF-2**α** binding in HIF-1**α** re-expressing cells demonstrated strong co-variance with HIF-2**α** binding in control cells (Fig 1B, compare vectors HIF-2**α**(VA) and HIF-2**α**(1**α**RE)). These analyses suggest that HIF-2**α** binding is largely unaffected by HIF-1**α** re-expression. To test this more quantitatively, we compared the relative strength of HIF-2 binding signals in HIF-1**α** re-expressing, versus control cells, across the 1719 endogenous HIF-2**α** binding sites identified in the control cells. This analysis revealed a tight correlation centered on equity of binding in the two cell lines, for both HIF-2**α** binding and for HIF-1**β** binding (S4 Fig). Thus, under these conditions, HIF-1**α** re-expression does not appear to globally antagonize HIF-2**α** by disrupting HIF-2**α** binding, either through direct competition for binding sites, or by competing HIF-1**β** away from HIF-2**α**.

### Extensive binding of re-expressed HIF-1α at new functional sites

In contrast to limited effects on existing HIF-2 binding, re-expression of HIF-1**α** led to extensive new binding across the genome, with specific HIF-1**α** signal identified at 5147 sites. Marked correlation was observed between HIF-1**α** and HIF-1**β** binding in these HIF-1**α** re-expressing cells (Fig 1A, compare iv and v). In keeping with this, the PCA confirmed strong covariance between HIF-1**α** and HIF-1**β** in HIF-1**α** re-expressing cells indicating that these sites are largely distinct from those binding endogenous HIF-2**α** (Fig 1B, compare vectors HIF-1**α**(1**α**RE) and HIF-1**β**(1**α**RE) and contrast with HIF-2**α**(VA) and HIF-1**β**(VA)). Taken together, these findings indicate that following its re-expression HIF-1**α** binds to a large number of sites distinct from endogenous HIF-2**α** sites, most likely as a HIF-1**α**/HIF-1**β** heterodimer. Furthermore, total HIF-1**β** chromatin immunoprecipitation signal (number of reads mapping to peak regions) increased approximately 2–3 fold following re-expression of HIF-1**α** (also see S5 Fig). This suggests that HIF-1**β** protein abundance is not limiting for endogenous HIF binding in VHL-defective 786–0 cells, and that additional HIF-1**β** can be recruited to DNA binding sites following increased expression of a dimerization partner.

Analysis of the HIF-1**α** binding indicated that its pan-genomic distribution was strikingly non-random, being strongly enriched close to particular classes of gene promoter (Fig 1C and 1D) and closely resembling HIF-1**α**-specific patterns of binding in other cell types[10,20,22,39]. Annotation of the HIF-1**α** ‘nearest-neighbor’ promoters, according to transcript class, revealed that 68% are associated with protein-coding genes, with the remainder largely associated with long non-coding RNAs (lncRNAs) and antisense RNAs (Fig 1D); proportions that are similar to those reported for HIF-1**α** in MCF7 breast cancer cells[39].

We next sought to define the functional effects of this new HIF-1**α** binding on gene expression. Pan-genomic gene expression was profiled in control and HIF-1**α** re-expressing cells by RNA-seq. Genes were ranked according to a combination of fold-change in transcript level following re-expression of HIF-1**α** and the significance of this change[35]. Using this ranking, gene set enrichment analysis (GSEA)[34] of the nearest gene to each HIF-1**α** binding site demonstrated a highly significant (p<0.001) association between HIF-1**α** binding and positive, but not negative, regulation of these transcripts (Fig 1E). This is consistent with previous findings that HIF acts predominantly as a transcriptional activator[10,19]. It also indicates that the new HIF-1**α** binding is functional, and has direct and largely positive effects on expression across large numbers of genes.

In contrast, we observed few effects of HIF-1**α** re-expression on transcript levels of (nearest neighbor) HIF-2**α** binding genes. GSEA demonstrated a positive, but non-significant (p = 0.15) effect of HIF-1**α** re-expression on the expression of HIF-2**α** binding genes (S6 Fig), presumably as a direct effect of HIF-1**α** binding to some of these sites (Fig 3C). Importantly, there was no evidence of large-scale down-regulation of HIF-2**α** binding genes (i.e. no enrichment of HIF-2**α** binding genes amongst the downregulated genes) as a consequence of HIF-1**α** re-expression in 786-O cells. This confirms the finding that re-expressed HIF-1**α** does not significantly antagonize HIF-2**α** activity across the genome.

### Overexpression of HIF-2α further activates its endogenous targets

Since overexpression of HIF-2**α** in 786-O cells has the opposite effect on tumor xenograft growth to re-expression of wild-type HIF-1**α**[11], we next examined the effect of increasing HIF-2**α** levels on HIF binding and gene expression. We first sought to distinguish whether binding of HIF-2**α** in VHL defective 786-O cells is saturated or whether overexpression of HIF-2**α** would increase binding at sites that are already occupied in control cells. To test this, we correlated the strength of HIF-2**α** binding signals in cells overexpressing transfected HIF-2**α** with that in the control cells. This revealed an increase in both HIF-2**α** signals and HIF-1**β** signals at the majority of endogenous HIF-2**α** binding sites following HIF-2**α** overexpression (S7 Fig) indicating that endogenous levels of HIF-2**α** in 786–0 cells are not sufficient to fully load these HIF-2**α** binding sites.

### Extensive non-canonical binding of overexpressed HIF-2α

**Fig 2:**
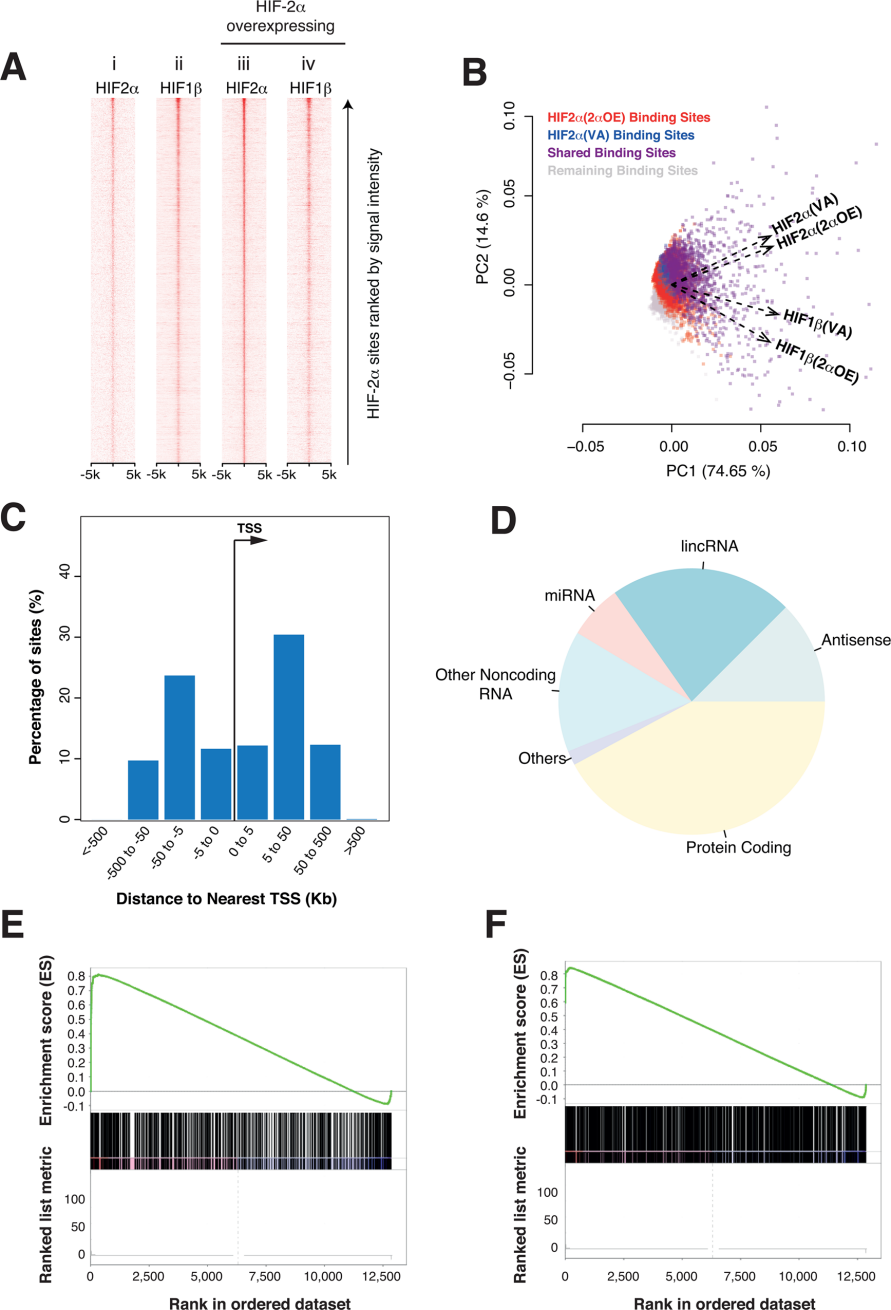
HIF-2α overexpression in 786-O cells. (A) HIF-2**α** binding sites in the HIF-2**α** overexpressing cells were identified by peak calling and ranked on the vertical axis according to signal intensity. Heat maps of these sites (±5kb on horizontal axis) showing ChIP-seq read density for the indicated HIF subunits were generated for both the control cells (i, ii) and the cells with HIF-2**α** overexpressed (iii, iv). In contrast to re-expressed HIF-1**α**, overexpressed HIF-2**α** binds to a large number of sites (compare i and iii), without HIF-1**β** (compare iii and iv) and has little effect on the distribution of HIF-1**β** (compare ii and iv). (B) Biplot showing Principal Component Analysis (PCA) of ChIP-seq signal intensity (RPKM values) for both individual binding sites (dots) and HIF-subunits (vectors) across all HIF-binding sites identified in control cells and in HIF-2**α** overexpressing cells. Sites binding endogenous HIF-2**α** in control cells are shown in blue while sites binding re-expressed HIF-1**α** are shown in red, sites binding both are colored purple and the remaining sites are colored grey. PCA for HIF subunits shows that HIF-2**α** and HIF-1**β** co-vary more closely in the control cells (compare HIF2**α**(VA) and HIF1**β**(VA)) than in the overexpressing cells (compare HIF2**α**(2**α**OE) and HIF1**β**(2**α**OE)). (C) Histogram of the distance to nearest transcription start site (TSS) for HIF-2**α** binding sites in cells overexpressing HIF-2**α**. (D) HIF-2**α** binding sites in the HIF-2**α** overexpressing cells were categorized according to the class (Ensemble) of the nearest gene. The relative frequency of each class is shown by pie chart. Gene set enrichment analysis (GSEA) for the set of genes nearest to (E) HIF-2**α** binding sites in the control cells and (F) newly identified HIF-2**α** binding sites in the overexpressing cells, when genes are ranked according to fold-change and significance in mRNA expression following overexpression of HIF-2**α** (horizontal axis).

Remarkably however, pan-genomic analysis revealed a total of 5283 discrete HIF-2**α** binding sites in the 786–0 cells overexpressing HIF-2**α**. These sites include many of those occupied by endogenous HIF-2**α**. In contrast to the findings following re-expression of HIF-1**α**, heat map analysis of HIF-2**α** and HIF-1**β** binding indicated that many of these sites are occupied solely by HIF-2**α**, without HIF-1**β** (Fig 2A, compare iii and iv). Concordant with this, PCA analysis revealed that transfected HIF-2**α** and HIF-1**β** binding co-vary less extensively in cells overexpressing transfected HIF-2**α** than in the control cells (Fig 2B).

As with HIF-1**α** binding in re-expressing cells, the distribution of transfected HIF-2**α** binding sites was strikingly non-uniform across the genome (Fig 2C and 2D). Interestingly, despite the large increase in number of sites they conformed to a pattern similar to that observed for endogenous HIF-2**α** in both these (S8 Fig) and non-CCRC (MCF7) cells[10,39], but distinct from that for HIF-1**α**. In contrast with HIF-1**α**, the distribution was more promoter-distal (Fig 2C). Furthermore, as with the endogenous HIF-2**α** binding patterns, annotation of ‘nearest neighbor’ genes revealed that a substantially greater proportion were at non-coding gene loci, than was observed for re-introduced or endogenous HIF-1**α** (Fig 2D). These differences between HIF-**α** isoforms, which were observed irrespective of cell type expression level or number of sites bound, suggest that the ability to recognize promoter-distant enhancers at non-coding gene loci versus promoter proximal sites is an innate property of HIF-**α** isoforms.

Finally, we sought to identify whether increased loading of existing HIF-2**α** binding sites, or binding of HIF-2**α** at newly detected sites, or both, is associated with functional effects on gene expression. We used GSEA to test enrichment of HIF-binding loci (nearest-neighbor genes) amongst genes whose expression was changed by HIF-2**α** overexpression (as measured by RNA-seq). We performed this analysis for both the binding sites that were occupied by endogenous HIF-2**α**, and those that were newly observed following HIF-2**α** overexpression. An association with positive, but not negative, effects on gene expression was observed for both groups of binding loci (Fig 2E and 2F). Thus, constitutive activation of HIF-2**α** in 786–0 cells is submaximal and increasing HIF-2**α** leads to both quantitative and qualitative effects on HIF target gene expression.

Interestingly, close inspection of the apparently new binding sites frequently revealed low levels of HIF binding at these sites in the control cells, which were below the thresholds for identification of significant binding, but above those observed at a set of control regions (S9A Fig). This suggests that these signals are in fact ‘real’ and reflect uneven loading of potential HIF binding sites (S9B Fig).

### Analysis of HIF-2α and HIF-1α binding motifs

**Fig 3:**
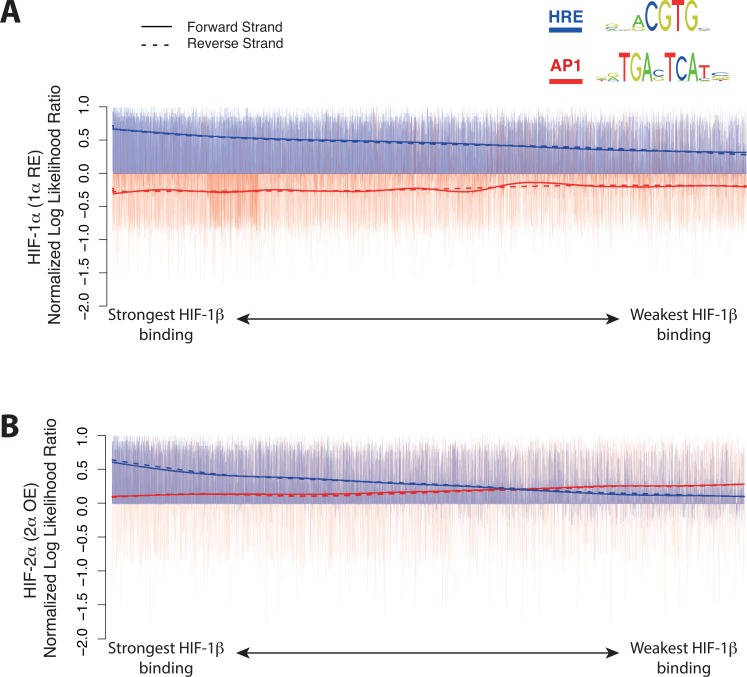
Preferential distribution of AP-1 binding motifs at HIF-2α versus HIF-1α binding loci. In addition to a hypoxia response element (HRE) motif, analysis of sites binding endogenous and overexpressed HIF-2**α** also identified an AP-1 motif. For each site, the maximum normalized log likelihood ratio for the AP-1 motif in red and the HRE motif in blue is plotted on the vertical axis as a bar chart. A smooth spline cubic fit line is overlaid to show the trend. The smoothing parameter is automatically determined using a ‘leave-one-out’ cross validation as implemented by the Smooth.spline function in R. Sites were categorized as binding (A) re-expressed HIF-1**α**, (B) overexpressed HIF-2**α** and ranked according to the HIF-1**β** signal at each site. Spline fit curves are overlaid (solid/dashed lines) to indicate overall trends across both forward and reverse strands. (A) Sites binding re-expressed HIF-1**α** show specific enrichment (positive score) for the HRE motif that decreases as the HIF-1**β** signal falls. In contrast, these same sites show depletion of the AP-1 motif. (B) Sites binding overexpressed HIF-2**α** show enrichment of the HRE motif that declines more steeply as the HIF-1**β** signal falls. In contrast to sites binding re-expressed HIF-1**α**, those binding overexpressed HIF-2**α** show enrichment of the AP-1 motif that increases (and exceeds that seen for the HRE) as the HIF-1**β** signal falls.

Since HIF-1 and HIF-2 are reported to recognize an identical core DNA binding sequence[10], it is unclear why binding of HIF-1**α** and HIF-2**α** is distributed so differently. To address this we started by analyzing the immunoprecipitated sequences for transcription factor binding motifs using MEME-ChIP. As expected, the canonical HRE motif (Jaspar/MA0259.1)[40] was enriched in all sets of binding sites; those occupied by endogenous HIF-2**α**, overexpressed HIF-2**α** and re-expressed HIF-1**α** and enrichment was positively correlated with the strength of the HIF-1β signal (Fig 3A and 3B and S8C Fig, blue line). The second most enriched motif was an AP-1 (TRE) binding motif (Jaspar/MA0491.1)[40]. Interestingly, this enrichment was quite differently distributed between HIF-1**α** and HIF-2**α** binding loci. Strong enrichment was observed in sequences binding overexpressed HIF-2**α** (Fig 3B). However in marked contrast to the HRE motif, in the binding set for over-expressed HIF-2**α**, enrichment of the AP-1 motif was inversely correlated with the strength of HIF-1**β** binding (Fig 3B). Furthermore, in the set of HIF-1**α** binding loci, the AP-1 binding motif was significantly depleted (Fig 3A). Overall the differential distribution of this AP-1 motif between HIF-1**α** and HIF-2**α** binding loci was highly significant (p < 10^−16^). Taken together these findings suggest that the AP-1 binding motif is important in the differential distribution of HIF-1**α** versus HIF-2**α** binding sites across the genome at least in this setting.

### Direct HIF-isoform-specific transactivation is associated with opposing prognosis in human kidney cancer

Many studies reported overall associations between HIF-dependent gene expression and adverse prognosis in cancer (reviewed in[3,41]), but there is little data that relates prognosis to HIF-isoform specific transcriptional targets and to isoform-specific HIF-binding patterns. We therefore sought to explore associations between HIF isoform-specific targeting defined in 786–0 cells, and genes expression patterns associated with clinical prognosis derived from The Cancer Genome Atlas (TCGA- https://tcga-data.nci.nih.gov/tcga/) [6].

We first stratified 415 CCRC patients, for whom both clinical data and tumor gene expression data were available, into good and poor prognosis groups based on a series of commonly used clinical criteria (survival, histological grade, metastasis, age)[42], (S10A Fig). From this stratification we ranked each gene, based on the significance and fold-difference in its median expression level, between the good and poor prognosis groups. We then sought to determine how HIF-**α** isoform specific changes in gene expression in 786–0 cells associated with this rank using GSEA. These analyses revealed striking isoform specificity in the associations. Genes that were upregulated by HIF-1**α** re-expression in 786-O cells were enriched (p = 0.01) amongst those genes that were more highly expressed in the good prognosis tumors (S10B Fig). Conversely, genes that were downregulated by HIF-1**α** were enriched (p<0.001) amongst those genes more highly expressed in the poor prognosis tumors (S10C Fig). Comparable analysis for HIF-2**α** regulated genes revealed a non-significant tendency for HIF-2**α** up-regulated genes to be more highly expressed in poor prognosis tumors, and for HIF-2**α** down-regulated genes to have a lower expression in poor prognosis tumors (S10D and S10E Fig). Taken together, these results indicate that the HIF-**α** isoform specific interventions in 786-O cells are relevant to clinical CCRC, and support the existence of opposing effects of HIF-1**α** and HIF-2**α** in a clinical context.

**Fig 4:**
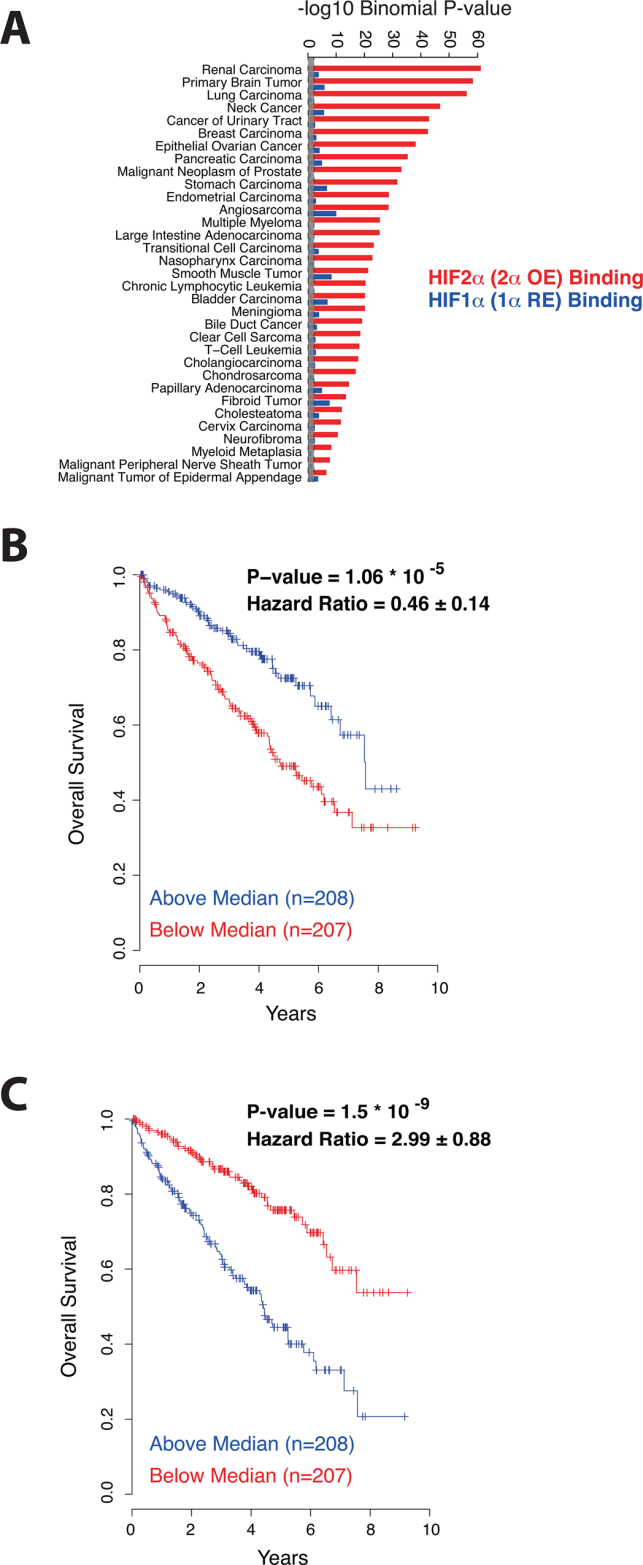
HIF-1α and HIF-2α binding genes confer opposing prognosis in kidney cancer. (A) The genes nearest to re-expressed HIF-1**α** (blue bars) and overexpressed HIF-2**α** (red bars) binding sites were defined and examined for enrichment amongst genes annotated in different cancers using the Human Disease Ontology database (http://www.disease-ontology.org).–log10 Binomial p-values are plotted for each set of HIF-binding genes in each type of cancer. Grey bar denotes p = 0.05 (-log10, 1.3) level of significance. HIF-2**α** nearest binding genes are consistently more significantly enriched amongst cancer-associated genes than are HIF-1**α** binding genes. (B) Differential HIF-1**α** binding genes or (C) differential HIF-2**α** binding genes were filtered for significant associations with overall survival and used to generate a weighted gene predictor of prognosis for each set of genes. Patients were then divided into those with above or below median values for each gene predictor and subjected to Kaplan-Meier survival analysis. The Cox proportional hazard model indicated a significant protective effect for patients with above median gene predictor values based on the HIF-1**α** binding genes. Conversely, patients with above median values for the HIF-2**α** binding gene predictor had a significantly worse prognosis.

Since the differential gene expression observed in 786-O cells is determined at least in part by disparate HIF-**α** subunit binding, we next sought to relate these differences in binding to cancer pathways. Gene ontology annotation of genes neighboring HIF-binding sites revealed a strong bias towards cancer association for HIF-binding genes, which was stronger for HIF-2**α** than HIF-1**α** associated transcripts (Fig 4A), in keeping with the pro- and anti-tumorigenic actions of HIF-2**α** and HIF-1**α** in this setting.

We next examined whether differential HIF-binding in 786-O cells could provide a predictive model of patient prognosis[37,38] in the TCGA CCRC dataset. First, genes with overlapping HIF-1**α** and HIF-2**α** binding (defined by signal intensities within a 3-fold difference) and those lacking a significant individual (univariate) association with prognosis were excluded. In total, 93 genes (44 in the HIF-1**α** binding group and 49 in the HIF-2**α** binding group) showed individual associations with prognosis that were significant. For each HIF-**α** isoform there was a mixture of genes associated with both a favorable and a poor prognosis. However, combined gene signatures generated for either the HIF-1**α** or HIF-2**α** binding genes were both significantly associated with overall patient survival (Fig 4B and 4C and S11 Fig). Specifically, the HIF-1**α** binding gene signature was associated with higher gene expression in good prognosis patients (Fig 4B and S11A Fig) and conversely that generated for the HIF-2**α** binding genes showed higher expression in the poor prognosis patients (Fig 4C and S11B Fig). These findings indicate that although HIF-1**α** and HIF-2**α** transcriptional targets may have heterogeneous effects on tumor behavior, the overall balance of HIF-1**α** transcriptional targets favors a good prognosis, while the balance of HIF-2**α** targets confers a poor prognosis.

## Discussion

Our findings reveal remarkable complexity in the HIF transcriptional response and its relationship to VHL-associated CCRC. Despite an identical consensus recognition sequence[10], large numbers of sites with distinct HIF-isoform specific binding preferences were defined. Our data suggests that it is this, rather than competition for binding at the same chromatin sites, or competition for dimerization with HIF-**β**, that underlies the contrasting HIF-isoform specific effects on experimental and clinical CCRC. It also points to a role for other transcription factors such as AP-1 in directing differential pan-genomic patterns of HIF-**α** binding, at least in this setting. Therefore differences in HIF-binding patterns and/or additional transcriptional activator activity may (at least in part) explain the different effects of HIF-1 and HIF-2 in different types of cancer.

Several systematic differences were observed in patterns of HIF-**α** binding. First, although as expected, HIF-**α** binding was generally associated with HIF-1**β** binding, when overexpressed, this was more robust for HIF-1**α** than HIF-2**α**, which appeared to be capable of binding without HIF-1**β**, particularly in association with AP-1 motifs. This suggests that non-canonical interactions of HIF-2**α** with AP-1 may be important[43], as has been reported for the interaction of HIF-**α** with Myc proteins[44,45]. Further analysis will be required to understand the precise nature of such interactions and their relationship to the biology of CCRC. However it is of interest that pVHL-inactivation has been reported to activate AP-1, in part due to up-regulation of an atypical protein kinase c-JunB pathway[46] and that CCRC-specific regions of accessible chromatin are highly enriched for an AP-1 motif[47]. Second, distinct pan-genomic patterns of HIF-**α** binding were observed that conform strikingly to those previously observed in other contexts. HIF-2**α** bound more distant from promoters and was more likely to be associated with non-coding RNAs than HIF-1**α** binding, the extent of bias being similar to that recently been described for the endogenous HIF-**α** proteins in MCF7 breast cancer cells[10,39]. This suggests that these patterns reflect some intrinsic property of the protein rather than expression level or cellular context.

A somewhat surprising finding in this work was the much larger number of discrete HIF-**α** binding sites observed after re-expression of HIF-1**α** or enhanced expression of HIF-2**α**, as compared to previous reports of the pan-genomic distribution of HIF binding by our laboratory and others[10,20,22,48,49]. Close inspection of these apparently new binding sites frequently revealed low, sub-threshold, levels of HIF ChIP-seq signal, which likely reflect uneven loading of potential HIF binding sites. This raises the interesting possibility that uneven binding site loading during graded hypoxic induction may be important in shaping the HIF response. Further work will be required to explore the physiological consequences of this in normal and cancer contexts.

The associations between binding of HIF-**α** in 786–0 cells and prognostic patterns of gene expression in human CCRC, clearly supports the relevance of these patterns to the clinical behavior of CCRC. Clear overall associations between HIF-1**α** binding and genes specifying good prognosis were mirrored by associations between specific HIF-2**α** binding and genes specifying poor prognosis, supporting the hypothesis that differential HIF-**α** binding patterns are driving contrasting effects on CCRC outcomes. Importantly however gene specific analyses revealed considerable heterogeneity in these effects. Individual gene analyses revealed that both the HIF-1**α** and the HIF-2**α**-associated gene-predictor each encompassed genes associated with both good and bad prognosis, indicating that each HIF-**α** isoform could potentially activate a spectrum of pro- and anti-tumourigenic effects. Given the prevalence of hypoxia and upregulation of the HIF system in cancer, understanding the factors that control, and potentially re-balance, HIF-**α** target gene repertoires as cancer develops will clearly be of importance.

## Supporting Information

S1 Fig Expression levels of endogenous and transfected HIF-1α and HIF-2α in 786-O cells. (A) Normalized read counts (RPKM–Reads Per Kilobase per Million reads) showing endogenous and transfected mRNA levels for HIF-1**α** (red) and HIF-2**α** (blue). Note endogenous HIF-1**α** is truncated (see S3 Fig), whilst re-expressed HIF-1**α** is full-length. (B) Immunoblot of HIF-1**α**, HIF-2**α** and **β**-actin in 786-O cells infected with control and HIF-**α** expressing viruses and in RCC4 cells, which express full-length HIF-1**α**. Quantitation of band intensities revealed HIF-2**α** protein levels to be roughly 8-times higher in the HIF-2**α** infected cells than in the control cells and HIF-1**α** protein levels to be 15-20-fold higher in the HIF-1**α** infected cells when compared to RCC4 cells.
(EPS)

S2 Fig Binding of endogenous HIF-2α and HIF-1β in control 786-O cells. (A) Scatterplot showing good correlation between the intensity of ChIP-seq signal (RPKM count) for HIF-1**β** (y-axis) and that for HIF-2**α** (x-axis) at the endogenous HIF-2**α** peaks. (B) Spatial distribution of HIF-2**α** and HIF-1**β** ChIP-seq signal (RPKM values) centred on the endogenous HIF-2**α** peaks (maximum HIF-2**α** signal ± 1kb) showing that HIF-2**α** and HIF-1**β** signals co-localize precisely.
(EPS)

S3 Fig 786-O cells express truncated HIF-1α, which is able to bind DNA. (A) RNA-seq alignment (red) of HIF-1**α** transcripts in control 786-O cells showing read density across the gene (annotated in blue). The schematic (black) relates the genomic and domain structures of HIF-1**α** (bHLH–basic helix-loop-helix domain, PAS–PER, AHR, ARNT, SIM domain, N–N-terminal transactivation domain, ID–inhibitory domain, C–C-terminal transactivation domain). The RNA-seq demonstrates an absence of reads mapping to exons 13, 14 and 15 encoding the C-terminal activation domain. Exons 11 and 12 encoding the N-terminal activation domain are greatly reduced in read number. In addition, there is abnormal splicing of intron 10, and therefore any transcripts containing exons 11 and 12 will be incorrectly translated C-terminal to exon 10. Paired-end mating of reads (black dotted lines) confirms the existence of fusion transcripts containing both HIF-1**α** and SNAPC1 sequences, presumably as a result of deletion of the HIF1A gene transcriptional termination site.
(EPS)

S4 Fig Re-expressed HIF-1α does not antagonize HIF-2α binding. (A) A scatter plot showing HIF-2**α** ChIP-seq signal (RPKM counts) at sites binding endogenous HIF-2**α** in the control cells. HIF-2**α** signal intensity in cells re-expressing HIF-1**α** (y-axis) is plotted against that for HIF-2**α** in the control cells (x-axis). The majority of HIF-2**α** signals in both the control and HIF-1**α** re-expressing cells correlate positively and are clustered around the (dotted) line representing equal HIF-2**α** binding in the two conditions. (B) A scatter plot showing HIF-1**β** signals at the same sites in both HIF-1**α** re-expressing and control cells. Similar to HIF-2**α**, HIF-1**β** binding signals show a positive correlation between the two conditions clustered around the (dotted) line representing equal HIF-1**β** binding in the endogenous and HIF-1**α** re-expressing cells.
(EPS)

S5 Fig The effect of HIF-1α re-expression on HIF-1β binding distribution. The scatter plot shows ChIP-seq signal (RPKM count) for HIF-1**β** in the HIF-1**α** re-expressing cells (y-axis) versus HIF-1**β** in the control cells (x-axis) at sites binding endogenous HIF-2**α** in the control cells (blue) and at sites binding re-expressed HIF-1**α** (red). The HIF-1**β** signal shows a clear separation between the HIF-2**α** and HIF-1**α** binding sites. Specifically HIF-1**α** re-expression increases the HIF-1**β** signal at sites binding re-expressed HIF-1**α** (i.e. the red dots cluster above the dotted line representing equity of binding), whilst at the sites binding endogenous HIF-2**α** in the control cells, the HIF-1**β** signal was little altered by HIF-1**α** re-expression (i.e. the blue dots are clustered around the dotted line representing equal binding).
(EPS)

S6 Fig Re-expressed HIF-1α does not globally antagonize transactivation of HIF-2α binding genes. Gene set enrichment analysis (GSEA) for the set of nearest-neighbour genes that are closest to the endogenous HIF-2**α** binding sites in control cells. All genes are ranked according to their fold-change and significance following re-expression of HIF-1**α** (horizontal axis). Genes nearest to endogenous HIF-2**α** binding sites are enriched amongst the genes upregulated by re-expressed HIF-1**α**, but there is little enrichment amongst the genes downregulated by re-expressed HIF-1**α**.
(EPS)

S7 Fig Binding of endogenous HIF-2α in control cells is not fully saturated. (A) A scatter plot showing HIF-2**α** ChIP-seq signal (RPKM counts) at sites binding endogenous HIF-2**α**. The signal intensity in cells expressing transfected HIF-2**α** (y-axis) is plotted against that in the control cells (x-axis). HIF-2**α** binding signal intensity is positively correlated under the two conditions, and is generally increased in cells expressing transfected HIF-2**α** (i.e. the majority of signals cluster above the dotted line representing equal binding). (B) A similar plot showing HIF-1**β** ChIP-seq signal at the same set of binding sites exhibiting a comparable increase in HIF-1**β** binding in cells expressing transfected HIF-2**α**.
(EPS)

S8 Fig Binding distribution of endogenous HIF-2α in control 786-O cells. (A) Histogram of the distance to nearest transcription start site (TSS) for HIF-2**α** binding sites in control cells. (B) HIF-2**α** binding sites in the control cells were categorized according to the class (Ensemble) of the nearest gene. The relative frequency of each class is shown by pie chart. (C) Analysis of sites binding endogenous HIF-2**α** identified an AP-1 motif as well as the HRE motif. For each site, the maximum normalized log likelihood ratio for the AP-1 motif in red and the HRE motif in blue is plotted on the vertical axis as a bar chart. Sites were ranked according to the HIF-1**β** signal at each site. Spline fit curves are overlaid (solid/dashed lines) to indicate overall trends across both forward and reverse strands. Enrichment of the HRE motif decreases and enrichment of the AP-1 motif increases as the HIF-1**β** signal falls.
(EPS)

S9 Fig Low-level binding of endogenous HIF-2α at sites binding over-expressed HIF-2α. (A) Sites binding HIF-2**α** in the overexpressing 786–0 cells were ranked (x-axis) according to HIF-2**α** signal intensity (displayed in green on y-axis). These same sites were then examined in the control 786–0 cells for binding of endogenous HIF-2**α** (ranked on the x-axis and displayed in blue on the y-axis). Although the endogenous HIF-2**α** signals were lower than those for transfected HIF-2**α**, signals below the peak-calling threshold were observed at these sites in the control cells. To determine whether these were above background levels, we used ENCODE Faire-seq data from 786-O cells (GSM1011120) to randomly select an identical number of open chromatin sites (excluding HIF-2**α** sites). These sites were also ranked (x-axis) according to their endogenous HIF-2**α** binding signal (displayed in red on the y-axis). The binding signal for endogenous HIF-2**α** in control cells was considerably greater at sites identified as binding HIF-2**α** in the overexpressing cells than at the randomly selected accessible sites. This indicates that the majority of newly detected HIF-2**α** binding sites in HIF-2**α** overexpressing cells are in fact weak HIF-2**α** binding sites in the control cells. (B) Sites binding any isoform across all the datasets were ranked according to HIF-2**α** RPKM counts in control cells (indicated by the red line). The signal intensity for HIF-2**α** in the overexpressing cells was then plotted (blue dots) together with a spline smoothed (blue) trend line. When the signal intensity in the overexpressing cells is compared with the control cells, HIF-2**α** overexpression is seen to lead to a global but uneven increase of the HIF-2**α** signal. Specifically, there was a greater fold-increase in binding amongst the weaker sites than was observed amongst the stronger sites.
(EPS)

S10 Fig Expression of HIF-1α transcriptional targets correlates with clinical prognosis in CCRC. RNA-seq data from 415 patients studied by The Cancer Genome Atlas (TCGA) was analysed as described in methods. Based on clinical criteria, patients were divided into two prognostic groups illustrated in (A) a Kaplan-Meier plot of overall survival for each group (red = good prognosis, blue = poor prognosis). Genes were then ranked according to the expression fold-difference and significance (see Eq 3 in methods) between the good prognosis tumours and the poor prognosis tumours. (B) GSEA for the set of genes induced by re-expressing HIF-1**α** in 786-O cells showed that they were generally more highly expressed in the good prognosis tumours. (C) GSEA for genes suppressed by transfected HIF-1**α** in 786-O cells revealed that they were more highly expressed in the poor prognosis tumours. The same analysis was performed for (D) genes induced and (E) genes suppressed by overexpressed HIF-2**α** showing an opposing, although non-significant association.
(EPS)

S11 Fig HIF-1α and HIF-2α binding gene predictors. The genes within 10kb of both differential (> 3-fold difference in intensity) HIF-1**α** and HIF-2**α** binding sites were tested using Cox proportional hazard model for their ability to predict patient prognosis. Genes with a p-value of <0.05 in the univariate analyses were then used to generate a multivariate predictor for both the HIF-1**α** and HIF-2**α** as described in the methods. The genes and their respective weights as fitted by the gene predictor model are shown for (A) a HIF-1**α** predictor and (B) a HIF-2**α** predictor. The blue bars show genes upregulated in good prognosis patients and the red bars show genes upregulated in bad prognosis patients.
(EPS)
